# Effect of a Pedometer-based Exercise Program on Cancer Related Fatigue and Quality of Life amongst Patients with Breast Cancer Receiving Chemotherapy

**DOI:** 10.31557/APJCP.2020.21.6.1813

**Published:** 2020-06

**Authors:** Aagna Gandhi, Stephen Rajan Samuel, K Vijaya Kumar, PU Prakash Saxena, Prasanna Mithra

**Affiliations:** 1 *Department of Physiotherapy, Kasturba Medical College, Mangalore, Manipal Academy of Higher Education, India. *; 2 *Department of Radiation oncology, Kasturba Medical College, Manipal Academy of Higher education, Mangalore, India. *; 3 *Department of Community Medicine, Kasturba Medical College, Mangalore, Manipal Academy of Higher Education, Manipal, India. *

**Keywords:** Breast cancer, fatigue, pedometer, quality of life

## Abstract

**Background:**

Breast cancer is the most common cancer amongst Indian women. Cancer treatments leads to various side effects out of which Cancer-Related fatigue (CRF) is one of the most under-addressed side-effects. It is experienced the most in patients receiving chemotherapy. Exercise has been proven to be a beneficial intervention to manage CRF but the benefits of pedometer-based exercise programs is under-studied in patients with breast cancer. Hence, we set out to investigate the effects of a pedometer-based exercise program for patients with breast receiving chemotherapy.

**Methods:**

The current study was a non-randomized controlled trial with 22 patients each in exercise and control group. The exercise group received a pedometer-based walking program, whereas the control group received standard physical activity advice. Fatigue, quality of life, functional capacity and body composition were assessed at baseline, 3rd week and 7^th^ week.

**Results:**

At the end of 7 weeks intervention, functional capacity, quality of life and skeletal mass were found to have improved with statistical significance, while the fatigue and changes in total fat did improve but were not statistically significant.

**Conclusion:**

A 7-week pedometer-based exercise program improved functional capacity, quality of life and percentage of skeletal mass and also shows to have prevented deterioration in fatigue levels in patients with breast cancer receiving chemotherapy.

## Introduction

Representing almost a quartile of all cancers, breast cancer is one of the most prevalent female cancer worldwide. Epidemiological data of 2012 revealed that cervical cancer is the most occurring cancer in Indian women, but according to the recent report on epidemiology of breast cancer in Indian women done in 2017, the number is now surpassed by the incidence rates of breast cancer and it being the major cause of cancer mortality (Malvia et al., 2017).

The primary treatment plans for patients with breast cancer including surgery, radiotherapy or chemotherapy when used solely or in combinations, have increased the survival rates in this population. However, it has led to an exponential rise in the number of cancer survivors globally. These treatment modalities have negative impact on the performance of daily activities due to their common side effects like nausea, vomiting, loss of appetite, fatigue, hair loss, diarrhoea and associated infections. Out of these, one of the major unaddressed and overlooked side effects of all the anti-cancer treatment is fatigue (Kapoor et al., 2015).

Cancer Related Fatigue (CRF) varies with the type of treatment received by the patients. A study carried out in India found that fatigue, as stated by patients undergoing anti-cancer treatments, reported in chemotherapy was 98.30% followed by chemo-radiotherapy and radiotherapy which was 78.57% and 45% respectively (Karthikeyan et al., 2012).

The pro-inflammatory state during chemotherapy results in fatigue, reduced physical activity and poor appetite that are the classical signs of cancer cachexia which has a major impact on the change in body composition, especially on body fat percentage and skeletal mass throughout the course of treatment (Kleckner et al., 2019). This is clinically important, because weight and muscle loss are a primary concern for these patients and directly impacts their functional capacity.

Physical activity programs can help reduce these cancer-related and treatment-related side effects and increase physical function and fitness (Kokts-Porietis et al., 2019). Less than 15% of breast cancer survivors have been reported to meet physical activity guidelines (150 min/ week of moderate-vigorous physical activity). The existing evidence suggests that patients having breast cancer with higher physical activity levels post-diagnosis have less chances of relapse and breast cancer-related mortality when compared to those with lower physical activity levels (Kokts-Porietis et al., 2019).

To address low physical activity status, walking is a standard home-based advice given but often unmonitored and non-measurable. The use of physical activity monitors like pedometers, accelerometers and other wearable devices can help these patients in monitoring and quantifying their physical activity through the number of steps taken by them in a day. As per the WHO recommendations, 8,000-10,000 steps a day accounts for moderate to vigorous levels of physical activity (Bauman et al., 2008; Møller et al., 2015). However, there is lack of literature regarding role of pedometer-based walking programs in patients with breast cancer receiving chemotherapy in addition, to a lack of exercise-based research amongst cancer survivors in India (Samuel et al., 2015). 

Therefore, this study was carried out to find the effect of a Pedometer-based program on CRF, quality of life (QoL), body composition and functional capacity in patients with breast cancer receiving chemotherapy.

## Materials and Methods

The study was a non-randomized controlled trial which was conducted from December 2018 to March 2020 amongst 44 breast cancer patients aged 18 years and above, seeking care and scheduled for chemotherapy at Kasturba Medical College Hospital, Mangalore, attached to Kasturba Medical College, Mangalore, Manipal Academy of Higher Education, Manipal, India. Approvals were obtained from the Institutional Scientific Review Committee and Institutional Ethics Committee (IEC) of Kasturba Medical College, Mangalore. The study was registered with Clinical Trial Registry of India (CTRI –CTRI/2019/11/022085). 

Patients with Eastern Cooperative Oncology Group (ECOG) (Oken et al., 1982) Score < 2 who were planned to start with Chemotherapy were included in the study. Cardiac clearance, which included ECG and ECHO, was taken prior to the start of chemotherapy. Patients with poor cardiac history or abnormal reports including platelet count < 80,000/µl of blood, haemoglobin < 8g/dl, severe orthopaedic and neurological problems and those who have any contraindications for exercise testing and prescription were excluded from the study. The subjects diagnosed with breast cancer scheduled for chemotherapy were approached and explained about objectives of the study in a language they understood and the willing participants signed a written informed consent. Depending on the inflow of the patients in hospital during the time frame of the study, all the eligible subjects (n=44) were recruited into this study where in 22 subjects were allocated to the exercise and control group each through 1:1 allocation method.

The patients were then assessed and baseline readings were taken for all the outcome measures before starting chemotherapy. Both the groups were assessed for fatigue using the Brief Fatigue Inventory (BFI) Scale (developed by MD Anderson Cancer Center), QoL using the Functional Assessment in Cancer Therapy- Breast (FACT-B) (Oliveira et al., 2014) scale and body composition using the Omron Karada Scan (HBF 375) Bio-electrical Impedance Analyser (BIA) (Lukaski et al., 1986) machine. The study group was given an Omron Pedometer (HJ-321) (Bassett and John, 2010) and the study participants were explained about the functioning of the same and diary to record the steps taken every day of the week. They were also explained that they can terminate the session if they observe any adverse symptom like dyspnoea, excessive fatigue, palpitations etc. 

The exercise group received a target of 10,000 steps to complete for 5 days a week which is a moderate intensity aerobic exercise (Bauman et al., 2008). The number of steps taken by the patient on day 1 was recorded and patient was asked to increase the number of steps by 5% each week which was confirmed through a phone call. The Pedometer had to be mounted throughout the day except while bathing and sleeping. The trial was a 7- week long intervention. The protocol was given along with the standard hospital care during the course of chemotherapy. The patients in the control group received Physical activity recommendations that is three 10-minute walks during the day for five days a week which is 150mins/ week as recommended by NCCN guidelines (Berger et al., 2018). Both the groups were assessed for the outcome measures at baseline and at 3rd and 7th week when their successive chemotherapy cycles were planned. 

The collected data was coded and entered onto IBM SPSS Statistics for Windows, Version 25.0. Armonk, NY: IBM Corp. Intention to treat analyses were performed. Results were expressed as proportions and summary measures (median with inter quartile range) using appropriate tables. For pair-wise comparisons across the groups, Mann Whitney U test adjusted for alpha error and for paired data, Wilcoxon Signed Rank test were used. A ‘p’ value of <0.05 was considered statistically significant.

## Results

Among the total of 44 study participants, there were no adverse events or protocol deviations during the course of the study. Of the 44 patients studied, 2 were men. Both the groups were comparable at baseline with regard to their age, gender and ECOG scores, as shown in Table 1. Standard chemotherapy regimen followed in the hospital included 4 cycles of 3 weekly doxorubicin with cyclophosphamide followed by 4 cycles of 3 weekly injection docetaxel. Demographics: Table 1 given further shows the characteristics of the patients taken at baseline

Fatigue: Fatigue was assessed using the Brief Fatigue Inventory Scale which has 9 questions and a maximum score of 90. The control group at baseline showed a median score of 3 which at the end of 3 weeks increased to 11 and further increased to 23 at the end of 7^th^ week. Whereas, the exercise group median score which was 6 at baseline by the end of 3^rd ^week and 7^th^ week increased to 4 and 11 respectively as shown in Table 2. Increase in fatigue was observed in both the groups, but the control group showed more increase in fatigue than the exercise group. The difference within the groups was found to be statistically significant (p<0.0001). However, the difference between the intervention and control groups was not found to be statistically significant (p=0.206). 

Quality of Life: QoL was assessed using Functional Assessment in Cancer Therapy-Breast (FACT-B) Scale. Control group showed a decrease in QoL by 41.9% with a median score of 121.50 (71.50, 127.75) at baseline and 65 (40, 78.50) at the 7th week, whereas the exercise group showed an increase in the QoL by 25% with a median score of 65 (39.75, 80) and 102 (76.25, 127.25) at baseline and 7^th^ week respectively as shown in Table 3. The control group showed statistically significant decrease in QoL (p < 0.0001) and the exercise group showed statistically significant increase in QoL (p < 0.0001). Also, the between group analysis (at the end of 7 weeks) showed statistically significant difference in the QoL signifying the positive impact of exercise on this important outcome measure in cancer survivors (p< 0.0001).

Functional Capacity: The functional capacity of all the recruited patients was assessed by a 6 min-walk test and using the distance covered as a parameter to interpret functional capacity. The distance covered by the control group decreased from baseline median score of 385 (357, 420) to 7^th^ week median score being 320 (310, 350) as shown in Table 4. The reduction is distance is 65 meters. On the other hand, the distance covered by the exercise group increased from baseline median score of 370 (345, 390) to 7^th^ week median score being 405 (380, 437.50). The difference in the distance covered was 35 meters. Using Wilcoxon Signed Rank test the decrease in the results of control group is statistically significant with a p < 0.0001 whereas the result of exercise group shows a p value of 0.004.

Body Composition: The control group shows an increase in total fat (TF) and BMI by 0.70% and 0.30 difference in the median scores respectively, whereas the exercise group shows a decrease in both with a value of 0.60 and 0.10 in that order as shown is Table 5. Skeletal Mass on the other hand shows a statistically significant increase in the exercise group with a difference in median score of 0.75 (p < 0.0001) but shows a reduction of 0.20 with a p-value of 0.053 when pre- intervention and post-intervention medians were compared in the control group.

**Figure 1:**
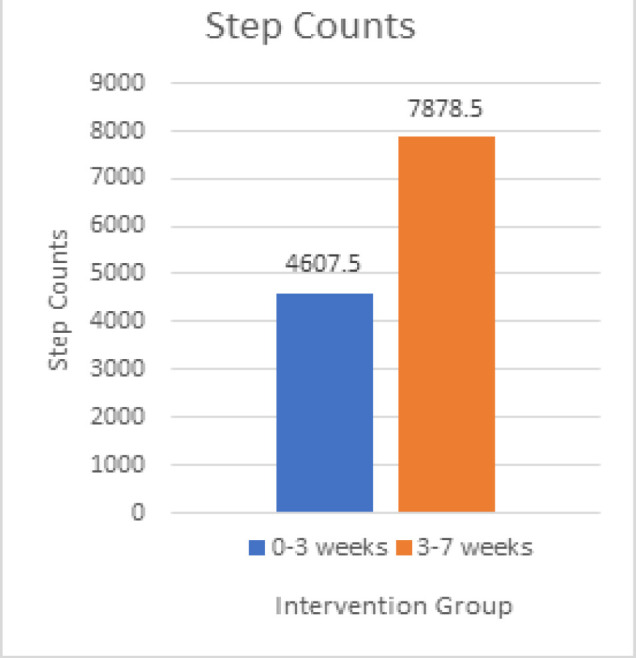
Change in Step-Counts Throughout the Study

Step Count: All the patients recruited in the exercise group received an exercise log where they had to enter the step count achieved by them every day and to mention if any adverse events were faced during the entire course of the study. The median score from baseline to 3rd week was 4607.50 (3797, 5560.50) and from 3rd week to 7^th^ week was 7878.50 (7013.50,8627.75) as shown in Figure 1. The results show a 32% increase in step-counts at the end of the study which was statistically significant with a p-value of less than 0.0001 using Wilcoxon Signed Rank Test.

**Table 1:** Baseline Characteristics of All the Patients Recruited in the Study

Characteristics	Control Group (n= 22)	Exercise Group (n= 22)
Age [Median (IQR)]	50 (47, 54.5)	50 (42, 54.2)
Gender [% within group]	Females 21 (95.5%)	Females 21 (95.5%)
	Males 1 (4.5%)	Males 1 (4.5%)
BMI [Median (IQR)]	22.8 (21.5, 24.5)	22.15 (19, 24.2)
ECOG [Median (IQR)]	1 (1,1)	1 (1,1)

n, represents number of patients; IQR, Inter-Quartile Range; BMI, Body Mass Index; ECOG, Eastern Cooperative Oncology Group.

**Table 2:** Results of Baseline and 7^th^ Week Brief Fatigue Inventory Scale Score of Control and Exercise Group

Timeline	Control Group (n=22)	Exercise Group (n=22)
Baseline Median (IQR)	3 (0, 11)	6 (2.5, 23)
7^th^ week Median (IQR)	23 (13, 29.5)	11 (6.5, 18.25)
*P*-value (within the group)	p < 0.0001 *	p < 0.0001 *

n, represents number of patients; IQR, Inter-Quartile Range; *, The result is significant at *p* < 0.0001

**Table 3:** Results of Pre- and Post- Intervention Scores of FACT-B Scale of Control and Exercise Group

Characteristics	Control Group (n=22)		*P*-value	Exercise Group (n=22)		*P*-value
	Pre	Post		Pre	Post	
	Median (IQR)	Median (IQR)		Median (IQR)	Median (IQR)	
FACT-B	121.5	65	p < 0.0001*	65	102	p < 0.0001*
	(71.50, 127.75)	(40, 78.50)		(39.75, 80)	(76.25, 127.25)	

n, represents number of patients; IQR, Inter-Quartile Range; *, The result is significant at p < 0.0001; Pre and Post, represents median scores of FACT-B scale at baseline and at 7^th^ week respectively.

**Table 4:** Results of 6-Minute Walk Test Pre- and Post- Intervention of Control and Exercise Group

Characteristics	Control Group (n=22)		*P*-value	Exercise Group (n=22)		*P*-value
	Pre	Post		Pre	Post	
	Median (IQR)	Median (IQR)		Median (IQR)	Median (IQR)	
6MWD	385	320	p< 0.0001*	370	405	0.004
	-357,420	(310, 350)		(345, 390)	(380, 437.50)	

n, represents number of patients; IQR, Inter-Quartile Range; *, The result is significant at p < 0.0001; Pre and Post, represents median scores of 6-Minute Walk Distance (6MWD) at baseline and at 7^th^ week respectively.

**Table 5:** Results of Changes in Body Composition parameters in Control and Exercise Group

Group	Categories	Pre	Post	Difference in Median	Significance (p-value)
		Median (IQR)	Median (IQR)		
Control Group (n=22)	Total Fat	36.50 (33.95, 42.37)	37.40 (34.75, 43.50)	0.70 ↑	0.001
	Skeletal Mass	22.50 (19.10, 25.90)	22 (18.65, 25.50)	0.20 ↓	0.053
	BMI	22.80 (21.50, 24.50)	23.20 (21.80, 24.90)	0.30 ↑	0.001
Exercise Group (n=22)	Total Fat	35.75 (32.70, 39)	35.20 (32.45, 37.52)	0.60 ↓	0.003
	Skeletal Mass	22.85 (19.77, 25.07)	23.10 (21.70, 26.27)	0.75 ↑	p < 0.0001*
	BMI	22.15 (19, 24.20)	22.15 (18.67, 24.97)	0.10 ↓	0.268

n, represents number of patients; IQR, Inter-Quartile Range; *, The result is significant at p < 0.0001; Pre and Post, represents median scores of body composition parameters at baseline and at 7^th^ week respectively; BMI, Body Mass Index

## Discussion

This trial studied the effectiveness of a 7-week Pedometer based walking program on fatigue, QOL, functional capacity and body composition during the first 3 cycles (Donovan et al., 2004) of chemotherapy in patients with breast cancer. A systematic review by Samuel et al., (2020) on the efficacy of pedometer-based walking program among the same type of population reported the evidence from 3 trials wherein majority of the findings were inclined towards psychological and dietary outcome measures.

Our study elucidates a statistically significant difference between the exercise and the control group in their QOL and functional capacity scores at the end of intervention period. This finding is of significance as an inexpensive device like pedometer can help attenuate loss in functional capacity and QOL in this population. Although components like fatigue did not show a between group statistically significant difference the increase in fatigue levels of the control group patients seen in this study is noteworthy while the decrease in fatigue levels of the exercise group which was significant for within group analysis is encouraging. 

Another finding of our study is the decrease in total fat in the exercise group and the increase in total fat of the control group which was found to be statistically significant in the between group analysis. This result suggests that exercise may prevent chemotherapy related weight gain in women receiving chemotherapy for patients with breast cancer.

A longitudinal study indicated that sarcopenia is prevalent in Indian cancer patients receiving treatment (Chauhan et al., 2020). Concurrently, our study showed an increase in skeletal mass in exercise group which was statistically significant (p < 0.0001). Therefore, exercise could be used as an intervention to prevent sarcopenia in patients receiving cancer treatment. Our study, as a novel approach, incorporated more of functional and holistic outcomes in this patient population. In this trial the intensity of the program was set using rate of perceived exertion (RPE) which made it easier for the patients to quantify it at home and hence made the protocol was feasible and well-tolerated one by the patients with an adherence rate of 93.2%.

According to a study done by Antoniu et al., (2015), on various outcome measures used to assess fatigue states that fatigue score in BFI scale has to be at least 7 to be considered as significant which implies that in our study the fatigue scores in the control and exercise group were insignificant at the beginning of the study. Although there was an increase in fatigue values in both the groups at the end of 7^th^ week, the increase in the control group was much exaggerated and statistically significant, while the increase in fatigue scores in exercise group was minimal and statistically not significant. This shows that exercise can play an important role in preventing escalation in development of fatigue in patients with breast cancer receiving chemotherapy. Our study shows consistent result with the NCCN guidelines (Berger et al., 2018) which suggests exercise as one of the most effective non-pharmaceutical interventions to overcome cancer-related and treatment-related fatigue (Berger et al., 2018).

Similar findings of improved physical well-being were noted in a study done by Djuric et al., (2011) wherein they assessed the fatigue outcome through the subcomponent fatigue scores in FACT-B scale. They indicated that the supervised group receiving counselling for physical activity through telephonic conversations showed a significant improvement in fatigue levels in the patients with breast cancer undergoing chemotherapy.

Besides the changes observed in fatigue, there was a significant change observed in the QoL between the control and the exercise group. The control group which received a standard unsupervised physical activity advice had shown a 41.9% fall in the QoL when assessed with FACT-B scale whereas the exercise group that received a step target and a pedometer to supervise the same exhibited 25% improvement in quality of life after enrolling in the study. A pilot study done by Kokts-Porietis et al., (2019) on home based physical activity protocols in patients with breast cancer survivors wherein supervised exercise protocols were conducted for at least 12 weeks. The study also concluded that a structured and a supervised exercise program results in a substantial improvement in QoL of these patients when compared to the regular physical activity advice which is unsupervised.

The functional capacity in our study was assessed using 6MWT. The distance covered in 6MWT improved in the exercise group by 35m while the control group showed a decrease of 65m. There was a statistically signiﬁcant difference in 6MWD from baseline within the groups. Although 50m is the minimal clinically important difference (MCID) for 6MWT, multiple studies have concluded that patients with cancer might have a lower MCID (Samuel et al., 2013; Samuel et al., 2019). Given the fact that the original MCID for 6MWT was derived from COPD (Bohannon and Crouch, 2017) patients it will be interesting to know in future studies if patients receiving treatment for cancer have a different MCID in terms of 6MWT.

Lastly, the body composition parameters especially BMI, Skeletal Mass and total fat also show statistically significant findings when compared within the group. Multiple aerobic and resistance exercise-based studies have been done to assess the changes in body composition taking BMI, through height and weight and body fat, through waist-hip ratio, as a parameter. Our study included skeletal mass also as a parameter along with BMI and total fat. Skeletal mass showed a significant increase in the exercise group whereas, the control group showed a decrease in skeletal mass. Sarcopenia or muscle loss has been reported in Indian head and neck cancer patients receiving chemoradiation (Chauhan et al., 2020) whereas limited research is done on the same in patients with breast cancer and this study indicates that exercise can play an important role to manage sarcopenia. 

The findings of our study with regards to decrease in BMI is in line with a pilot randomized controlled trial done by Pelekasis et al., (2016). That showed a statistically significant decrease in the BMI of the exercise group that received a pedometer-based physical activity combined with diaphragmatic breathing exercise, dietary consultation and cognitive behavioural therapy (CBT). The decrease in BMI in our study was not statistically significant, which indicates that an integrative program including breathing exercises, diet counselling and CBT when given with physical activity recommendation may bring about better results in preventing weight gain in patients receiving chemotherapy. 

A qualitative review done by Inglis et al., (2019) states that chemotherapy leads to xerostomia, altered taste, nausea and vomiting which has a profound impact on body composition especially leading to sarcopenia and sarcopenic obesity that can intensify the perceived fatigue in patients with breast cancer. 

Physical activity gains in our study which was measured through a pedometer states that a walking program with a five percent increase in step counts to the patient’s baseline values was well-tolerated by the patients and exhibited a good adherence level. 

Our study concludes that a supervised home-based walking program using a simple wearable device like pedometer provides significant improvement in CRF and QoL. It also has a positive impact on functional capacity and helps in reducing loss of skeletal mass. This study was a non-randomized controlled trial where the allocation was not concealed and no blinding was done which were the limitations of the study. Future randomized trials are required to further authenticate our findings.

In conclusion, a 7-week pedometer-based exercise program improved functional capacity, QoL and percentage of skeletal mass and also prevented the increase in fatigue levels amongst patients with breast cancer receiving chemotherapy.
